# Effect of Viscosity Reduction by Rubber Organic Degradation Agents in High-Rubber-Content Asphalt

**DOI:** 10.3390/ma18245619

**Published:** 2025-12-15

**Authors:** Jingzhuo Zhao, Junchang Gao, Kuan Jiang, Dawei Dong, Xingjun Zhang, Yong Huang, Yiqing Wang, Zhao Wang, Fucheng Guo

**Affiliations:** 1 School of Ecology and Environment, Xinjiang University, Urumqi 830046, China; 13893329103@139.com; 2 Gansu Traffic Planning Investigation Designing Institute Co., Ltd., Lanzhou 730010, China; 3 Institute of Emergent Elastomers, School of Materials Science and Engineering, South China University of Technology, Guangzhou 511442, China; gaojunchang163@163.com; 4 Key Laboratory of Beijing City for Preparation and Processing of Novel Polymer Materials, Beijing University of Chemical Technology, Beijing 100029, China; jiangkuan851222@163.com (K.J.); beijing425@163.com (D.D.); 5 Gansu Provincial Highway Construction Management Group Co., Ltd., Lanzhou 730099, China; 13669382619@139.com; 6 State Key Laboratory of Organic-Inorganic Composites, Beijing University of Chemical Technology, Beijing 100029, China; 7 Huangpu Green Advanced Materials Technology Research Institute, School of Materials Science and Engineering, South China University of Technology, Guangzhou 510530, China; 8 College of Civil Engineering, Lanzhou Jiaotong University, Lanzhou 730070, China; fcguo@chd.edu.cn

**Keywords:** rubber powder, impregnation and diffusion, high-content rubber powder-modified asphalt, diphenyl disulfide

## Abstract

The increase in the viscosity of high-rubber-content asphalt modified with rubber powder at high temperatures leads to processing difficulties and drastic changes in physical properties, which have long been a challenge in the asphalt industry. Although viscosity reducers have shown great potential in addressing these issues, their mechanisms of action in high-rubber-content asphalt modified with rubber powder remain unclear. This study employs diphenyl disulfide (DD) as a viscosity reducer and elucidates its mechanism of action in high-rubber-content asphalt, which includes three stages: (1) dissolution and dispersion in the asphalt matrix; (2) impregnation into the crosslinked network of the rubber powder; and (3) de-crosslinking via active free radicals. By optimizing the pre-impregnation time (12 h), temperature (110 °C), and rubber powder particle size (160–180 µm), the dispersion of DD can be enhanced, thereby improving the processability of high-rubber-content asphalt modified with rubber powder. Compared to untreated asphalt, the optimized conditions result in a significant reduction in the crosslinking density of 50% and a substantial decrease in viscosity at 180 °C. This study provides new insights into the viscosity reduction of high-rubber-content asphalt modified with rubber powder and contributes to a deeper understanding of the mechanisms of viscosity reducers.

## 1. Introduction

The application of waste tire rubber in asphalt modification for road construction not only mitigates the escalating issue of petroleum resource depletion but also eradicates the “black pollution” associated with the indiscriminate disposal of waste tires. Additionally, the use of such rubber-modified asphalt significantly enhances road performance, service life, durability, and noise reduction effectiveness [1,2,3,4,5]. However, the modification of asphalt with rubber predominantly employs the “wet process” [6,7,8], where the typical addition level of rubber powder ranges between 15% and 24% [9,10,11,12]. Further increasing the rubber powder content leads to a significant rise in the high-temperature viscosity of the modified asphalt and abrupt changes in its overall physical properties, which in turn affect the workability during construction and the comprehensive physical performance of the pavement [13,14,15].

To address the issues associated with high incorporation levels of rubber powder, many researchers have investigated methods to increase the amount of rubber powder in asphalt. Wang et al. [16] successfully prepared modified asphalt with rubber powder content exceeding 40% using high-speed shearing, which exhibited excellent performance in terms of high and low temperature resistance, fatigue resistance, and aging resistance. Li et al. [17] prepared an environmentally friendly high-content pre-treated crumb rubber-modified asphalt using waste cooking oil for pre-treatment. The rubber content in the asphalt reached 20%. Compared with conventional asphalt, it exhibits significantly enhanced high-temperature rutting resistance and low-temperature crack resistance. In addition, some researchers have adopted the method of adding organic rubber degrading agents to the rubber powder–asphalt system to prepare high-content rubber asphalt through in situ viscosity reduction [17]. Xu et al. [18] used waste edible oil to reduce the rotational viscosity of high-viscosity rubber asphalt, thus improving the processability of high-content rubber powder-modified asphalt. Moreover, the use of this viscosity reducer improved the dispersion of crumb rubber in the asphalt and enhanced its high-temperature stability.

During the process of preparing rubber powder-modified asphalt, it is effective to use rubber organic degradation agents to reduce the viscosity in situ, which can produce high-content rubber powder-modified asphalt. Compared with the traditional “two-step method”, which involves first activating the rubber powder and then using it for asphalt modification, in situ viscosity reduction technology offers greater advantages in terms of overall cost and is more readily scalable for industrial use [19,20,21,22]. However, despite the numerous benefits demonstrated by in situ viscosity reduction technology in practical applications, research on the mechanisms of action of rubber organic degrading agents in the preparation of high-content rubber powder-modified asphalt remains relatively scarce.

The increase in the viscosity of high-rubber-content asphalt modified with rubber powder at high temperatures leads to processing difficulties and drastic changes in physical properties, posing a significant challenge in the asphalt industry. Although viscosity reducers have shown promise in addressing these issues, their mechanisms of action in high-rubber-content asphalt modified with rubber powder remain unclear [23]. This study aims to elucidate the mechanism of action of diphenyl disulfide (DD) as a viscosity reducer in high-rubber-content asphalt. By simulating the process of dissolution, dispersion, and impregnation diffusion by DD in high-rubber-content asphalt, this study provides a theoretical basis for the subsequent research and development of related technologies and products. The novelty of this work lies in the detailed investigation of the radical-induced de-crosslinking process and its impact on the rheological properties of rubberized asphalt, offering new insights into the optimization of rubber powder pre-treatment conditions for industrial applications.

## 2. Materials and Experiments

### 2.1. Materials

Matrix asphalt (AP) was Qilu 70A, produced by the Qilu Branch of China Petrochemical Corporation, Zibo, China; rubber powder (CR) was all-steel radial tire rubber powder (270–380 μm/212–250 μm/160–180 μm) from Jiangyin Anqiang High Wear-Resistant Powder Rubber Co., Ltd., Jiangyin, China; the organic viscosity reducer diphenyl disulfide (DD) was supplied by Acros Organics, Geel, Belgium; isoprene rubber (IR) and natural rubber (NR) were both provided by China Nantai Rubber Co., Ltd., Nantong, China; styrene-butadiene rubber (SBR, 1502E) was obtained from the Qilu Branch of China Petrochemical Corporation, Zibo, China; dicumyl peroxide (DCP), accelerator (CZ) and accelerator (NOBS) were purchased from Tianjin No. 1 Organic Chemical Factory, Tianjin, China; diphenyl ether was sourced from Shandong Taixing New Materials Co., Ltd., Shandong, China; zinc oxide (ZnO) was supplied by Longchang Chemical Co., Ltd., Taipei, Taiwan, China; stearic acid (SA) was acquired from Chongqing Changjiang Yangfan Chemical Co., Ltd., Chongqing, China.

### 2.2. Preparation of Vulcanized Rubber

Rubber powder, a finely ground vulcanized rubber obtained from the crushing and sieving of waste tires, comprises components such as rubber, carbon black, plasticizers, antioxidants, and vulcanizing agents. To eliminate the interference of carbon black, plasticizers, antioxidants, sulfur, and sulfur-containing proteins in natural rubber in the diffusion of DD, spherical vulcanized rubber (SR) specimens were first prepared and reserved for subsequent use.

In the preparation process, an overoxide vulcanization crosslinking agent was used in place of sulfur for the rubber vulcanization crosslinking reaction. Specifically, SBR (40 phr, parts per hundred rubber; hereinafter the same), IR (60 phr), and DCP (1 phr) were kneaded in an open mill, and the Mooney viscosity (${ML}_{{}^{\circ}C1+4}^{100}$) of the kneaded rubber was measured. For IR, the vulcanization temperature was set at 160 °C, and the vulcanization pressure was 15 MPa. The optimal vulcanization time T_90_ of the sample was determined using a rubber vulcanization tester (ZL-3001 moving matrix rheometer, Zhongli Instrument Technology Co. Ltd., Dongguan, China), and the rubber was then vulcanized on a flat vulcanizing machine according to the vulcanization time derived from the vulcanization characteristic curve, resulting in SR with a diameter of 20 mm.

Furthermore, to simplify the factors influencing the chemical regeneration of DD in vulcanized rubber, this study also designed and prepared vulcanized rubber (SSR), which was free of carbon black, plasticizers, and antioxidants. The formulation of SSR is shown in Table 1. The vulcanization temperature was 143 °C, and the vulcanization pressure remained at 15 MPa, with the rest of the preparation steps being identical to those for SR.

### 2.3. Study on the Impregnation and Diffusion of DD in Vulcanized Rubber

In order to investigate the role of DD in the preparation of high-rubber-content asphalt, this study utilized diphenyl ether as a solvent instead of petroleum asphalt to dissolve and disperse DD, thereby simulating the dissolution and diffusion of DD. Experiments were designed to examine the effects of the diffusion time and temperature, and relevant materials were prepared to clarify the potential mechanisms.

To investigate the effects of different insulation stirring durations on the impregnation and diffusion of DD in vulcanized rubber, we selected stirring times of 2, 4, 6, 8, and 10 h based on practical process considerations commonly used in industrial applications. SR was treated with these different stirring durations, and a series of samples were prepared, named 2hSR, 4hSR, 6hSR, 8hSR, and 10hSR. Taking the 2 h processing as an example for explanation, to begin with, 100 g of diphenyl ether was heated to 90 °C. Then, 0.6 g of DD was added and stirred at 200 rpm under insulation for 30 min. Subsequently, spherical vulcanized rubber SR (approximately 31.5 g) was added and stirred under insulation for 2 h. After stirring was stopped, the SR was removed and the solvent on its surface was wiped away with filter paper. The SR was then cut radially from the edge to the center into three rubber slices with a thickness of 3.3 mm, which were marked as the peripheral layer, middle layer, and core layer. These three rubber slices were placed into liquid nitrogen to be frozen and then broken at the center. The fracture surfaces were sputter-coated with gold. Finally, the content of sulfur (S) at the center of the fracture surface was tested using scanning electron microscopy–energy-dispersive spectroscopy (SEM-EDS).

In addition, to study the mechanism of action of DD under different temperatures of diphenyl ether (70 °C, 80 °C, 90 °C, and 100 °C, based on practical process considerations commonly used in industrial applications), a series of samples were prepared, and these samples were named 70 °CSR, 80 °CSR, 90 °CSR, and 100 °CSR. The preparation process was the same as above, except that the insulation stirring duration was 10 h.

To further investigate the effects of the penetration and dispersion degree of DD on the desulfurization degree of vulcanized rubber, samples SSR were treated with different durations, and the crosslinking density was tested using an NMR crosslinking density meter. The specific preparation process was as follows: 100 g of diphenyl ether was heated to 90 °C; then, 0.6 g of DD was added and stirred at 200 rpm under insulation for 30 min. Subsequently, spherical vulcanized rubber SSR (approximately 31.5 g) was added and stirred under insulation for 0 h, 4 h, 8 h, and 10 h. After this, the mixture was rapidly heated to 195 °C and stirred under insulation for 6 h. Stirring was then stopped, the spherical rubber SSR was removed, and the solvent on its surface was wiped away with filter paper. The SSR was cut radially from the edge to the center into five rubber slices with a thickness of 2 mm, which were marked as the peripheral layer, outer-mid layer, inner-mid layer, inner-core layer, and core layer.

### 2.4. Study on the Viscosity Reduction Effect of DD in High-Content Rubber Asphalt

**Figure 1:**
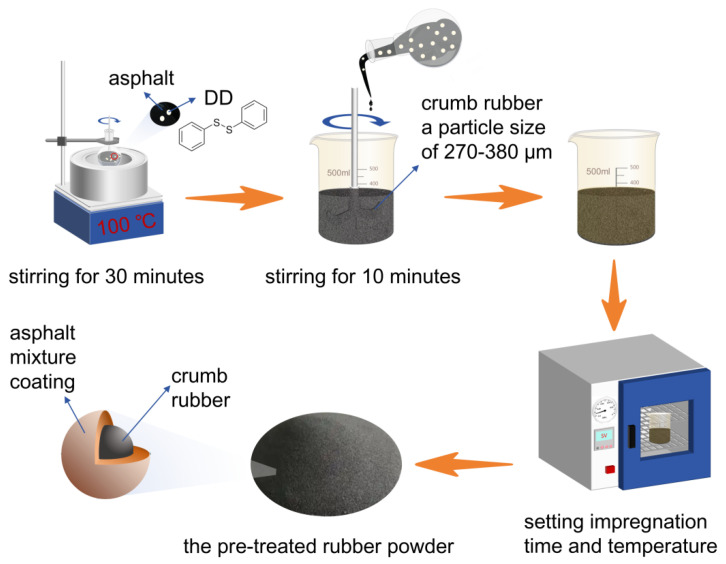
The process of pre-treating rubber powder.

To investigate the effects of the DD pre-impregnation time on the properties of rubber-modified asphalt, the following procedure was employed: 8 phr of asphalt was heated to 100 °C, and 0.6 phr of DD was added and stirred at 200 rpm for 30 min. Subsequently, 8.6 phr of this mixture was added to 35 phr of crumb rubber with a particle size of 270–380 µm. The mixture was then subjected to high-speed blending at 1200 rpm for 10 min using a high-speed mixer until the asphalt mixture was uniformly coated onto the rubber powder surface. The treated rubber powder was then placed in a vacuum drying oven at 90 °C for thermal treatment durations of 4, 8, 12, and 16 h. The process of pre-treating the rubber powder is shown in Figure 1. Thereafter, 92 phr of asphalt was heated to 195 °C, and 43.6 phr of the pre-treated rubber powder was added and stirred at 1000 rpm for varying durations to prepare rubber-modified asphalt samples.

To further explore the influence of the DD pre-impregnation temperature on the properties of rubber-modified asphalt, the vacuum drying oven temperature was set to 70 °C, 90 °C, 110 °C, and 130 °C to treat the rubber powder for 12 h, while all other steps remained consistent. Additionally, to study the effects of the rubber powder particle size on the properties of rubber-modified asphalt, experiments were conducted using crumb rubber with particle sizes of 160–180 µm, 212–250 µm, and 270–380 µm. The treatment temperature and time for the rubber powder were maintained at 195 °C and 12 h, respectively, while the remaining steps were kept identical.

### 2.5. Characterization

The spherical vulcanized rubber treated with DD was sectioned radially from the perimeter to the core into uniform-thickness slices. After being frozen in liquid nitrogen and fractured at the center, the fracture surfaces were sputter-coated with gold. The S element content at the center of the fracture surface was measured using the S-2501 SEM-EDS device from Cambridge Instruments, Cambridge, UK. Since the original rubber molecules contained no S element, as it existed only in DD molecules, the S element content could indicate DD’s penetration and diffusion in the original rubber.

The crosslinking density of vulcanized rubber comprises physical entanglement and chemical crosslinks. The chemical crosslinking density correlates with the extent of sulfur vulcanization. As for the crosslinking density of desulfurized reground rubber, it serves as an indicator of the rubber’s regeneration level. In this study, an MR-CDS 3500 NMR crosslinking density meter, manufactured by Germany’s Innovative Imaging Company (IIC Dr. Kuhn Innovative Imaging Corp. KG, Ettlingen, Germany), was employed to measure the crosslinking density of desulfurized rubber powder. The test conditions were set at a magnetic induction strength of 3.5 A/m, a frequency of 15 MHz, and a temperature of 60 °C.

In accordance with the JTG E20-2011 “Test Methods of Bitumen and Bituminous Mixture for Highway Engineering” standard [24], the physical properties of rubber-modified asphalt were tested using the following equipment. The LHDF-4 computerized asphalt softening point tester, the LHZR-5A computerized asphalt penetration tester, and the LHSY-1.5B low-temperature LCD extension tester from the Beijing, China, Lanhang Zhongke Testing Technology Research Institute were used to measure the softening point, penetration at 25 °C, ductility at 5 °C, and elastic recovery at 25 °C, respectively. The viscosity at 180 °C was measured using a coaxial cylinder geometry with the NDJ-1C Brookfield rotational viscometer from Shanghai, China, Changji Geological Instrument Co., Ltd. All tests were conducted under standard conditions to ensure the accuracy and reliability of the results.

## 3. Results and Discussion

### 3.1. Effects of Impregnation and Diffusion of DD in Vulcanized Rubber

#### 3.1.1. Effects of Impregnation Duration on DD Diffusion Efficiency

To quantitatively characterize the temporal evolution of DD diffusion from diphenyl ether into SR, the SR treated at different impregnation durations was named 2hSR, 4hSR, 6hSR, 8hSR, and 10hSR. The samples were radially microtomed into three concentric domains, identified as the peripheral-layer rubber, middle-layer rubber, and core-layer rubber. The S element content within each slice was used to gauge the extent of DD diffusion.

**Figure 2:**
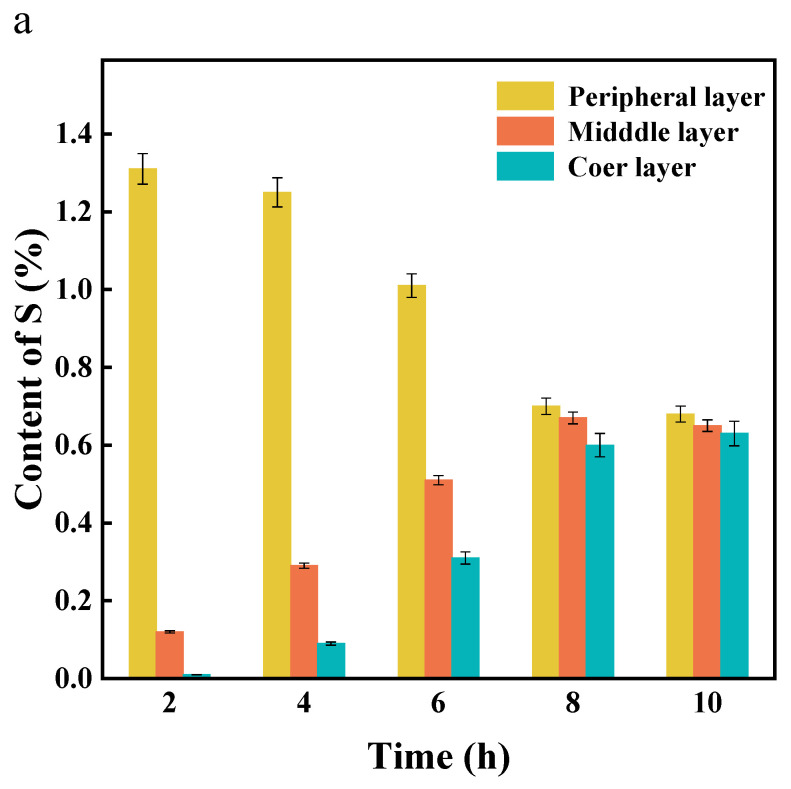

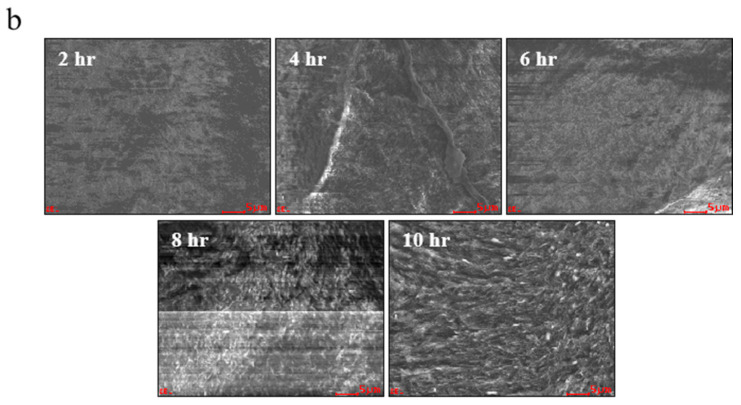
(**a**) The S element content with the impregnation duration in spherical vulcanized rubber; (**b**) SEM images of vulcanized rubber sections after different penetration durations.

As illustrated in Figure 2a, after 2 h of penetration, the sulfur content is predominantly localized in the peripheral layer of 2hSR, whereas the core layer exhibits the lowest sulfur content, indicating the incomplete inward diffusion of DD. With a prolonged impregnation duration, the sulfur content in the peripheral layer progressively declines, while that in the middle-layer rubber and the core layer increases accordingly, resulting in the continuous attenuation of the radial concentration gradient. By 8 h, the sulfur distribution across all layers converges to a nearly uniform value, signifying that DD’s impregnation has reached equilibrium. Extending the duration to 10 h yields no appreciable variation relative to 8hSR, confirming the establishment of a steady state. Collectively, the data demonstrate that DD penetration from diphenyl ether into the vulcanized rubber sphere follows a time-dependent equilibration process: an initially steep concentration gradient (high surface, low core) gradually flattens until homogeneity is achieved at approximately 8 h. Throughout this period, the total DD content within the sphere increases. The SEM images of the sample surface at different penetration times further confirm the DD penetration. Figure 2b demonstrates a progressive increase in structural looseness at the fracture surface of the vulcanized rubber with extended impregnation times, reaching equilibrium after 8 h. This phenomenon can be attributed to the gradual expansion of entangled polymer chains within the three-dimensional crosslinked network during impregnation [25].

#### 3.1.2. Effects of Impregnation Temperature on DD Diffusion Efficiency

**Figure 3:**
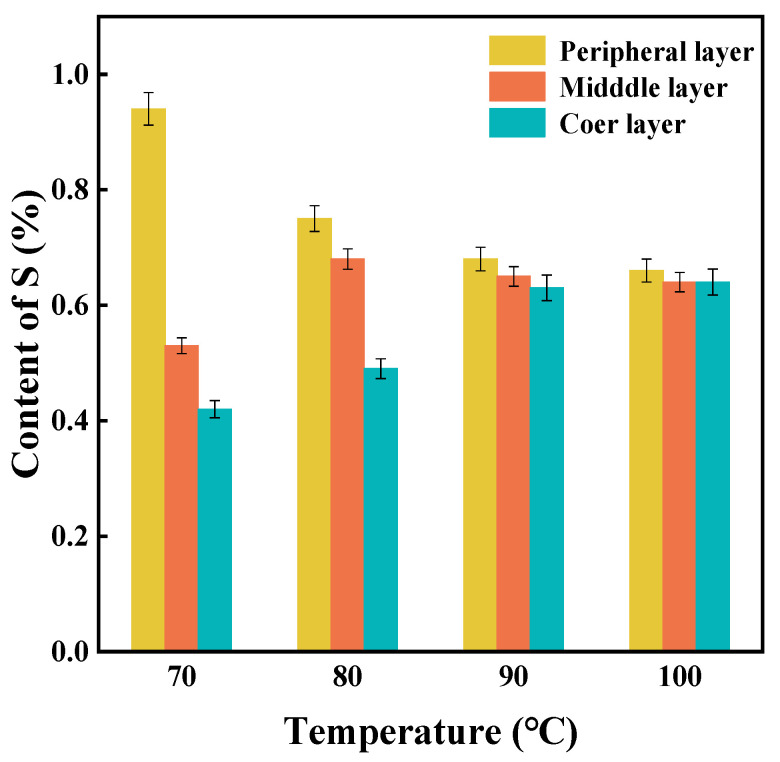
The S element content with the impregnation temperature in spherical vulcanized rubber.

Additional impregnation temperatures were systematically imposed to investigate the effects of the temperature on the diffusion behavior of DD into SR. As shown in Figure 3, the S element content of core-layer rubber increases with rising impregnation temperatures, in contrast to the S element content in middle-layer rubber and peripheral-layer rubber. Specifically, after the temperature reaches 90 °C, the content of the S element diffuses uniformly in SR. This phenomenon can be attributed to the enhanced thermal motion of DD molecules at elevated temperatures, facilitating their accelerated penetration into the rubber crosslinked network. It is worth noting that an increasing temperature also reduces the fractional free volume (FFV) of the vulcanized rubber [25,26]. At lower impregnation temperatures, due to the poor heat transfer of rubber, the reduction in FFV is less pronounced than the enhancement in DD molecular thermal motion, leading to an overall increase in the DD content within the rubber. As the impregnation temperature rises, a balance is eventually reached between the FFV reduction and the intensified thermal motion of DD molecules. Consequently, the DD content approaches saturation and achieves a uniform distribution.

#### 3.1.3. Effects of DD Impregnation and Diffusion on the Devulcanization of Vulcanized Rubber

In the field of waste rubber regeneration, the permeation diffusion degree of the rubber desulfurizer has a significant impact on rubber regeneration. Therefore, this work simulated the effects of the pre-impregnation and diffusion process of DD on the crosslinking density of rubber during the preparation of high-content rubber powder-modified asphalt and studied the effects of the pre-impregnation duration on the crosslinking density of vulcanized rubber. To eliminate interference from extraneous factors, a simplified spherical vulcanized rubber (SSR) was prepared without carbon black, plasticizers, or antioxidants. After DD treatment at various pre-impregnation durations, the SSR was microtomed into five concentric layers designated, from surface to center, as the peripheral, outer-mid, inner-mid, inner-core, and core layers.

**Figure 4:**
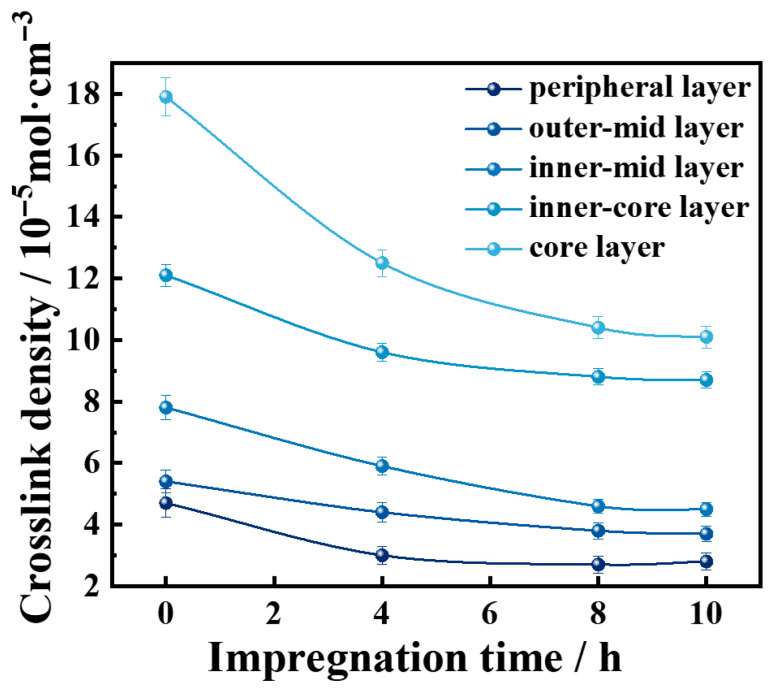
Time-dependent crosslink density curves of the simplified spherical vulcanized rubber with the impregnation duration.

As illustrated in Figure 4, after high-temperature devulcanization, the crosslink density in all regions of the SSR at 0 h was higher than that of samples treated for other impregnation durations. With a prolonged pre-impregnation duration, the crosslink density gradually decreased throughout the SSR after devulcanization and stabilized after 8 h. Among them, the content of the S element in the core layers decreased the most, with a reduction of more than 70%. Under the same pre-impregnation duration, the peripheral layer exhibited the lowest crosslink density, which progressively increased toward the core layer. This phenomenon can be explained by the fact that DD generates active free radicals at elevated temperatures, which subsequently initiate the cleavage of sulfur crosslinks, thereby reducing the crosslink density of the vulcanized rubber [27,28,29]. In the case of non-impregnation vulcanized rubber, due to the limited diffusion efficiency, the DD concentration decreases from the surface toward the core, resulting in a gradient reduction in available DD for devulcanization. Consequently, the extent of crosslink density reduction diminishes progressively from the surface to the interior. As the pre-impregnation duration increased, the DD distribution within the vulcanized rubber gradually approached diffusion radial uniformity. Moreover, owing to the poor thermal conductivity of rubber, the surface temperature remained higher than in the core under identical reaction conditions. Thus, the DD radicals at the surface exhibited higher reactivity, leading to more pronounced devulcanization at the surface. This explains the persistent non-uniformity in crosslink density reduction from the surface to the core.

### 3.2. Effects of DD Action on High-Rubber-Content Asphalt

The preceding experiments simulated the diffusion of DD into vulcanized rubber, elucidating the effect by which DD reduces the crosslink density. Under the combined influence of temperature and time, DD penetrates the rubber network, generates active radicals, and selectively cleaves sulfur crosslinks, thereby diminishing the crosslink density. To further clarify the viscosity-reducing mechanism of DD in high-rubber-content asphalt, this section presents experiments and their results.

#### 3.2.1. Effects of DD Pre-Impregnation Time on Rubberized Asphalt Properties

**Figure 5:**
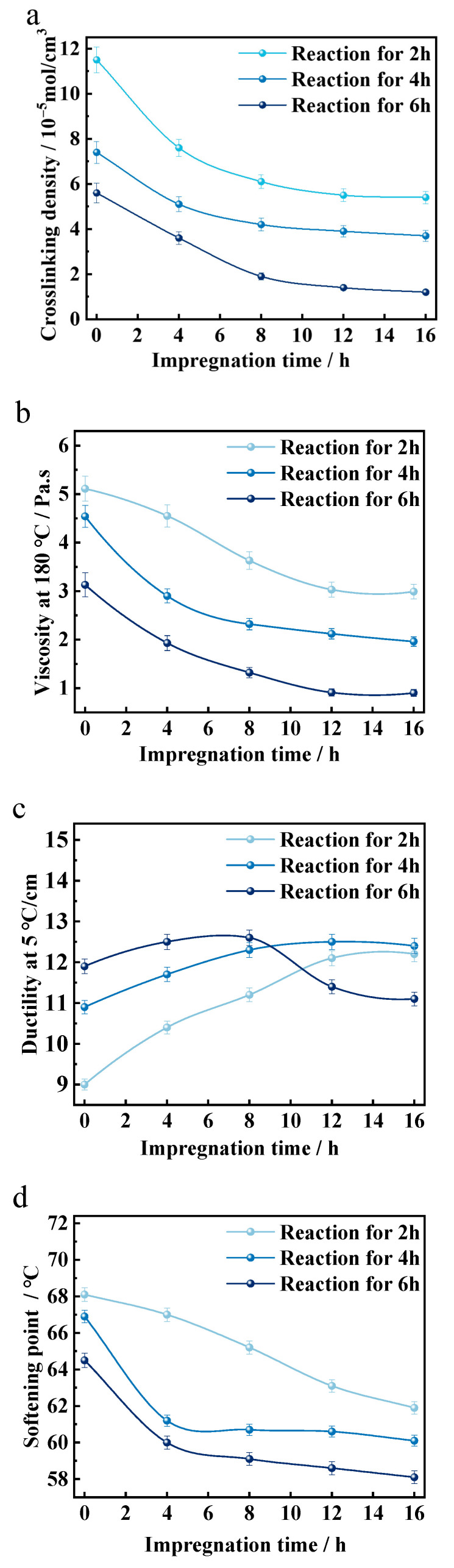

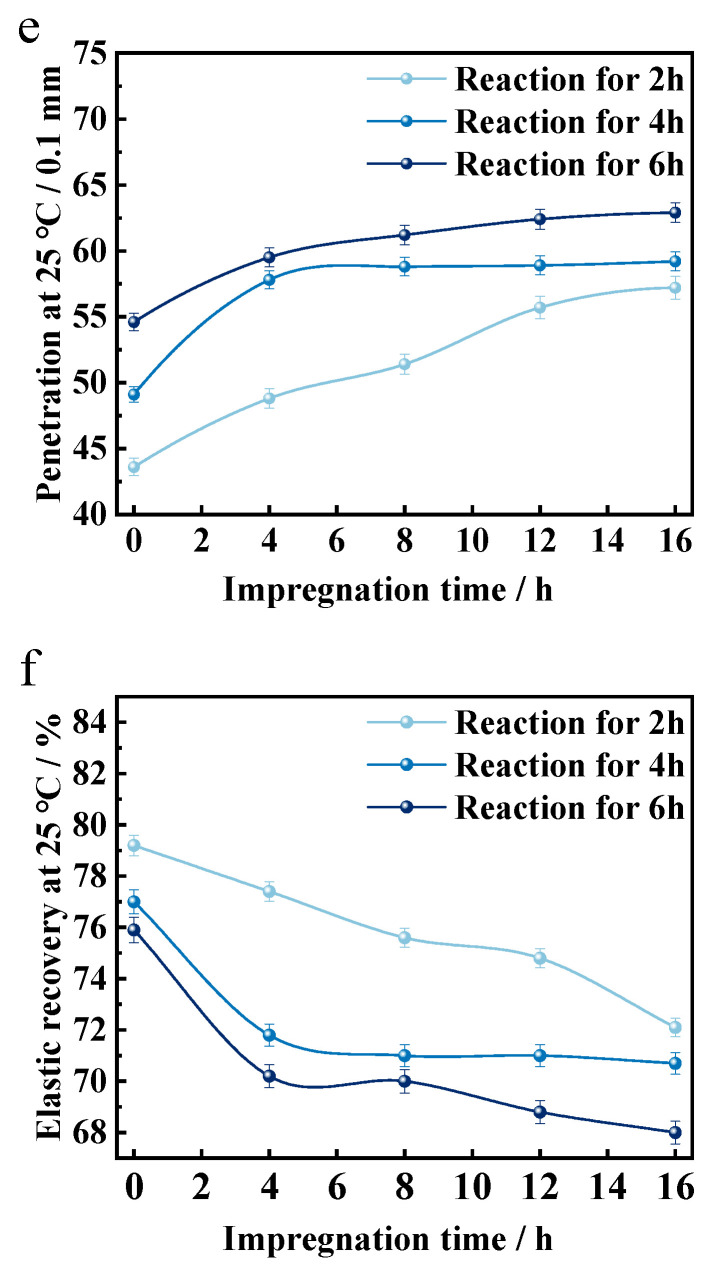
(**a**) The crosslinking density of desulfurized rubber powder. Physical properties of rubberized asphalt: (**b**) viscosity at 180 °C, (**c**) ductility at 5 °C, (**d**) softening point, (**e**) penetration at 25 °C (100 g, 5 s), (**f**) elastic recovery at 25 °C.

As shown in Figure 5, the rubber powder that was not subjected to pre-impregnation treatment exhibited the highest crosslinking density in asphalt. This high crosslinking density resulted in the significantly elevated viscosity of the rubberized asphalt at 180 °C, necessitating a longer development time to enhance its workability. With increasing pre-impregnation durations, the crosslinking density of the rubber powder in asphalt and the viscosity of the rubberized asphalt at 180 °C gradually decreased, stabilizing after 12 h. Compared to the original untreated rubber, the viscosity at 180 °C was reduced by 68.7%. Concurrently, the softening point of the rubberized asphalt decreased, while the penetration at 25 °C increased by 27.8%. However, the elastic recovery at 25 °C decreased. The ductility of the rubberized asphalt at 5 °C also varied with the pre-impregnation duration. When the pre-impregnation duration was less than 4 h, the ductility at 5 °C increased with the extension of the pre-impregnation time. In contrast, when the pre-impregnation duration exceeded 4 h, the ductility at 5 °C initially increased and then decreased. Further analysis revealed that, with the extension of the pre-impregnation duration, the crosslinking density of the rubber powder in asphalt and the viscosity of the rubberized asphalt at 180 °C continued to decline. When the pre-impregnation duration was less than 8 h, the ductility of the rubberized asphalt at 5 °C increased with the extension of the pre-impregnation duration. However, when the pre-impregnation duration exceeded 8 h, the ductility at 5 °C initially increased and then decreased. Meanwhile, the softening point of the rubberized asphalt continuously decreased, while the penetration at 25 °C continuously increased. The elastic recovery at 25 °C continued to decrease. These results indicate that pre-impregnation treatment significantly affects the rheological and mechanical properties of rubberized asphalt. The optimal pre-impregnation duration appears to be between 4 and 8 h, as this range provides a balance between reduced viscosity and improved ductility, while minimizing the negative impact on elastic recovery.

Overall, petroleum asphalt is generally considered to consist of components such as saturates, aromatics, resins, and asphaltenes [30,31,32]. Among these components, saturates and aromatics possess a good swelling capacity for vulcanized rubber [33]. By utilizing petroleum asphalt as the dispersing medium for DD and subjecting rubber powder to pre-impregnation and impregnation treatment, DD can be effectively dissolved and uniformly dispersed in petroleum asphalt. Under the influence of molecular thermal motion, as the pre-impregnation duration is extended, DD gradually permeates into and is uniformly dispersed within the rubber powder. After pre-impregnation treatment, as the development time of rubberized asphalt is prolonged, the rubber powder is heated from the outside to the inside. The DD, which is uniformly distributed within the rubber powder, forms active free radicals under the action of thermal energy. These free radicals react with the α-H atoms on the C=C double bonds of the vulcanized rubber, disrupting the three-dimensional crosslinked network structure and reducing the crosslinking density of the vulcanized rubber, thereby forming quasi-linear macromolecular fragments [28,34]. For rubber powder that has not undergone pre-treatment, the DD in asphalt gradually permeates into the interior of the rubber powder and simultaneously undergoes de-crosslinking reactions under the influence of heat and time. The extent and efficiency of these de-crosslinking reactions are lower compared to those of pre-treated rubber powder. This is reflected in the performance of rubber-modified asphalt, where the crosslinking density of the rubber powder in the rubberized asphalt is higher, and the high-temperature viscosity is greater. Consequently, a longer reaction time is required to meet the demands of production and construction.

Before DD reaches impregnation equilibrium, the amount of DD that permeates into the rubber powder increases with the extension of the pre-impregnation time of the rubber powder. At the same development time, the efficiency of DD in forming active free radicals and participating in de-crosslinking reactions is higher. Consequently, the de-crosslinking degree of the rubber powder in rubberized asphalt is greater, the crosslinking density is lower, the elasticity is weaker, and the content of quasi-linearized rubber macromolecular fragments is higher. In terms of rubberized asphalt performance, this results in lower high-temperature viscosity, a lower softening point, and lower 25 °C elastic recovery, while the 5 °C ductility and 25 °C penetration are higher.

When DD impregnation reaches equilibrium, it is uniformly dispersed within the three-dimensional crosslinked network of the rubber powder. Further extension of the pre-impregnation time of the rubber powder does not significantly change the de-crosslinking reaction degree of the rubber powder in rubberized asphalt at the same development time. As a result, the performance of rubberized asphalt remains essentially stable.

#### 3.2.2. Effects of DD Pre-Impregnation Temperature on Rubberized Asphalt Properties

**Figure 6:**
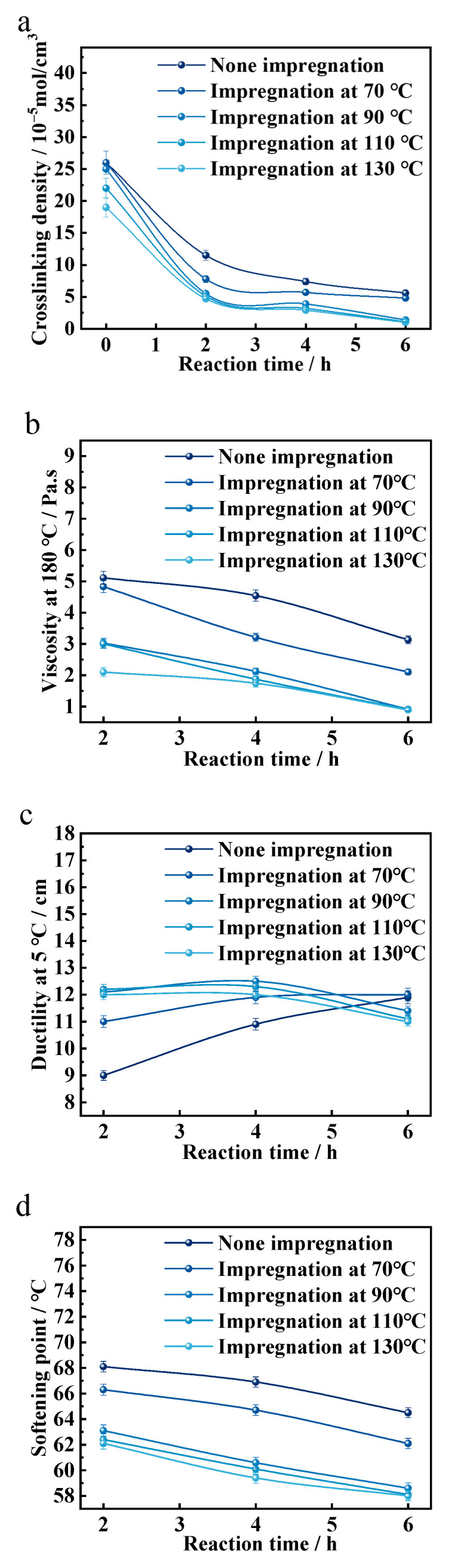

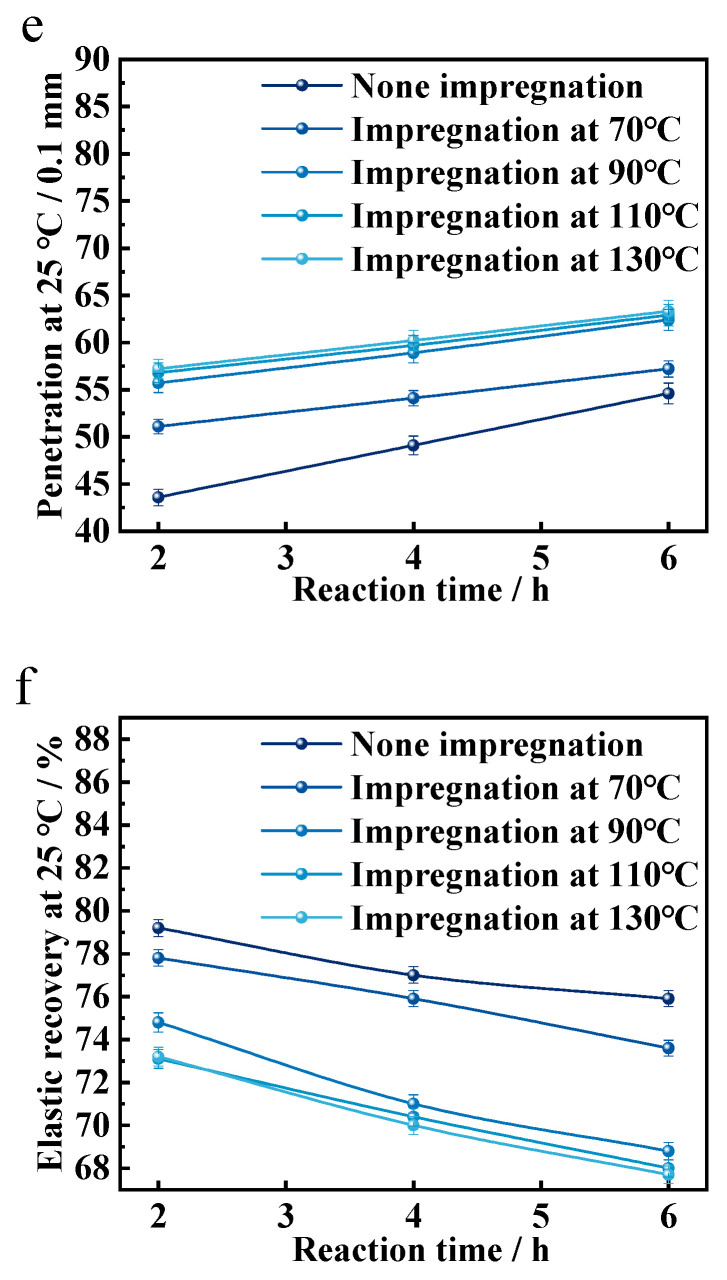
(**a**) The crosslinking density of desulfurized rubber powder. Physical properties of rubberized asphalt: (**b**) viscosity at 180 °C, (**c**) ductility at 5 °C, (**d**) softening point, (**e**) penetration at 25 °C (100 g, 5 s), (**f**) elastic recovery at 25 °C.

As shown in Figure 6, for rubber powder that has not undergone pre-impregnation treatment, the crosslinking density of the rubber powder in rubberized asphalt is the highest. With increasing pre-impregnation temperatures, the crosslinking density of the rubber powder in rubberized asphalt decreases. However, the degree of decrease becomes smaller when the pre-impregnation temperature exceeds 110 °C. The viscosity at 180 °C, softening point, and elastic recovery at 25 °C of the rubberized asphalt decrease, while the penetration at 25 °C increases. The ductility at 5 °C first increases and then decreases. As the development time is extended to 4 h, compared to the reaction at 0 h, with a pre-impregnation temperature of 110 °C, the crosslinking density of the rubber powder in rubberized asphalt decreases by 95.0%, the viscosity at 180 °C drops by 69.9%, the softening point lowers by 6.3%, the elastic recovery at 25 °C decreases by 2.4%, and the penetration at 25 °C increases by 5.5%. When the pre-treatment temperature is below 90 °C, the ductility at 5 °C increases. However, when the pre-treatment temperature exceeds 90 °C, the ductility at 5 °C first increases and then decreases.

As discussed in the previous sections, the impregnation and diffusion of DD into vulcanized rubber within a liquid medium are related to the temperature of the medium. The higher the temperature of the liquid medium, the stronger the thermal molecular motion of DD, resulting in a faster impregnation and diffusion rate and a shorter time required to reach swelling equilibrium. For rubber powder that has not undergone pre-impregnation treatment, the DD in asphalt gradually permeates into the interior of the rubber powder and undergoes de-crosslinking reactions under the influence of heat and time. However, the extent and efficiency of these de-crosslinking reactions are relatively low. After pre-impregnation treatment, as the development time is extended, the rubber powder is heated from the outside to the inside, leading to a greater extent and higher efficiency of de-crosslinking reactions. Consequently, the crosslinking density of the rubber powder after the reaction is lower, and the high-temperature viscosity of the rubberized asphalt is reduced. The time required to meet the workability conditions for production and construction is also shorter.

Before DD reaches pre-impregnation equilibrium and is uniformly dispersed, the higher the pre-impregnation temperature of the rubber powder, the greater the total amount of DD that permeates into the rubber powder, with a gradual decrease from the surface to the core. At the same reaction time, the efficiency of DD in forming active free radicals and inducing de-crosslinking reactions is higher. Consequently, the de-crosslinking degree of the rubber powder in rubberized asphalt is greater, the crosslinking density is lower, the elasticity is weaker, and the content of quasi-linearized rubber macromolecular fragments is higher. This is reflected in the performance of rubberized asphalt, where the viscosity at 180 °C, softening point, and elastic recovery at 25 °C are lower, while the ductility at 5 °C and penetration at 25 °C are higher.

Once DD pre-impregnation reaches equilibrium, further increasing the pre-impregnation temperature of the rubber powder allows DD to form a small amount of active free radicals. During pre-impregnation, these free radicals react with the three-dimensional crosslinked network of the rubber powder, causing de-crosslinking reactions and forming a small amount of quasi-linearized macromolecular rubber fragments. As a result, at the same reaction time, the overall physical properties of rubberized asphalt further deteriorate.

#### 3.2.3. Effects of Rubber Powder Particle Size on Rubberized Asphalt Properties

**Figure 7:**
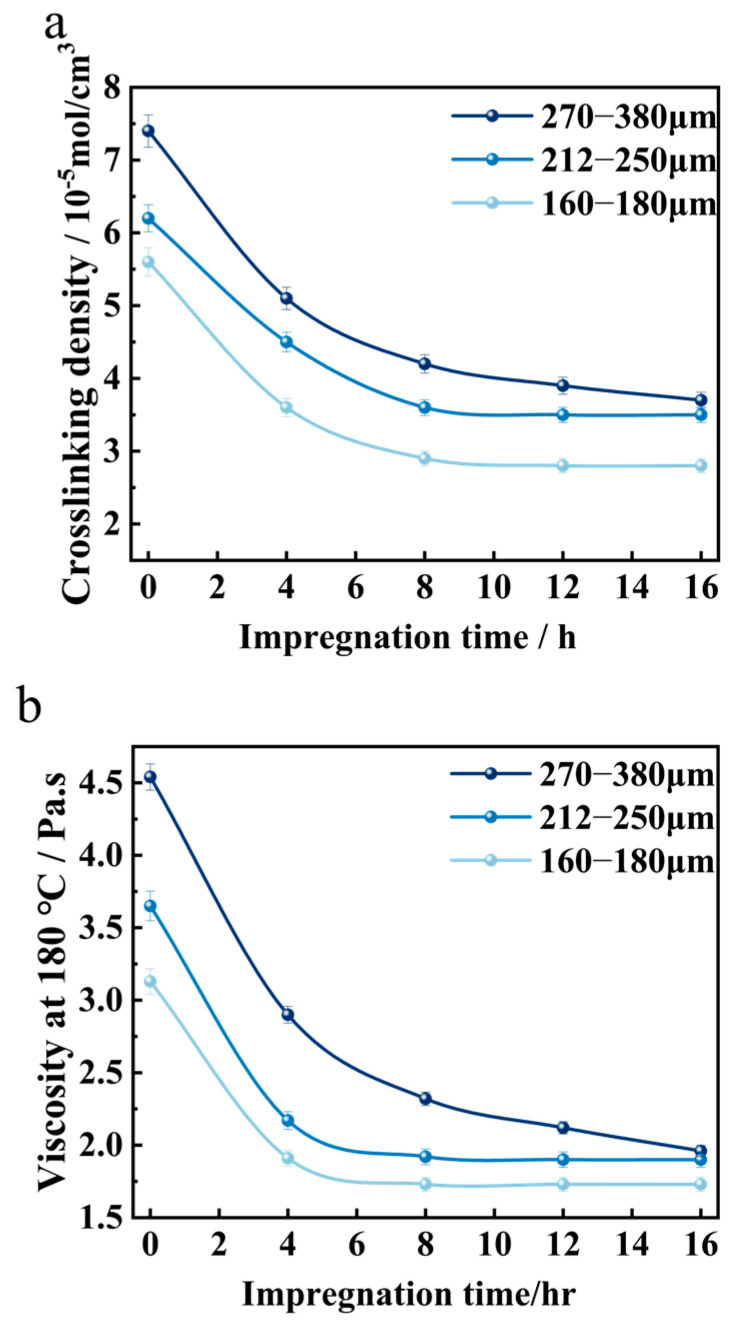

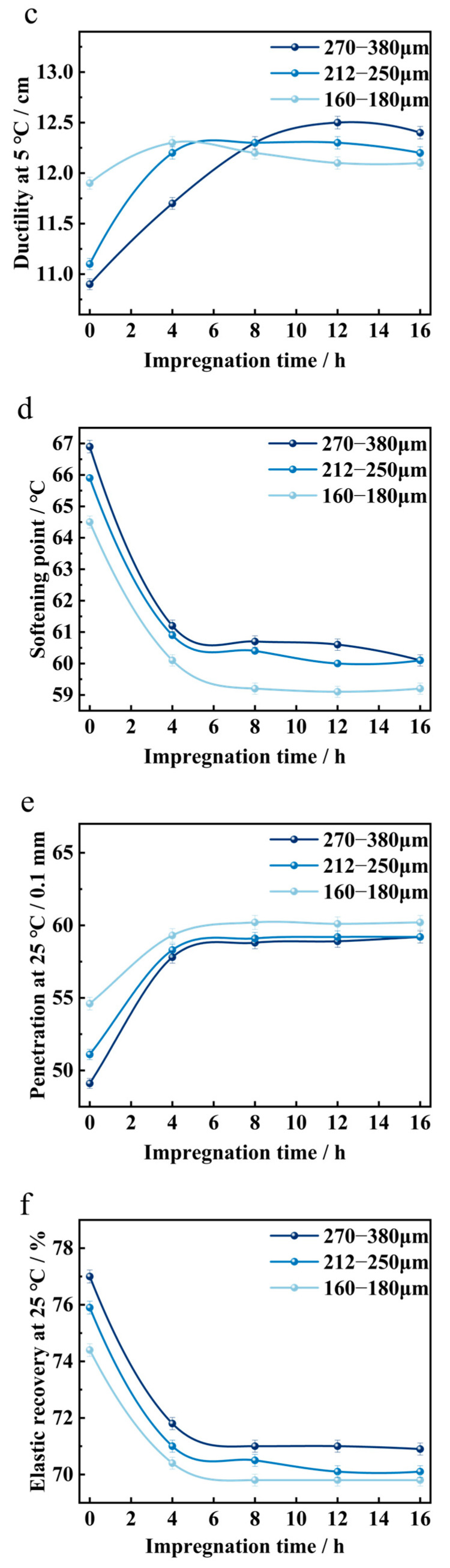
(**a**) The crosslinking density of waste rubber powder. Physical properties of rubberized asphalt: (**b**) viscosity at 180 °C, (**c**) ductility at 5 °C, (**d**) softening point, (**e**) penetration at 25 °C (100 g, 5 s), (**f**) elastic recovery at 25 °C.

As shown in Figure 7, in high-rubber-content asphalt modified with rubber powder, the rubber powder that has not undergone pre-treatment exhibits the highest crosslinking density after the reaction, and the viscosity of the rubberized asphalt at 180 °C is significantly elevated. With the extension of the pre-treatment time, the crosslinking density of the rubber powder after the reaction decreases. The smaller the particle size of the rubber powder, the shorter the swelling time required to reach equilibrium in the crosslinking density. Upon reaching a treatment duration of 8 h, compared to the untreated state at 0 h, the rubberized asphalt modified with 160–180 μm rubber powder exhibits a 44.7% reduction in viscosity at 180 °C. Additionally, the ductility of the rubberized asphalt at 5 °C rises by 2.5%, the penetration at 25 °C increases by 10.3%, the softening point decreases by 8.2%, and the elastic recovery at 25 °C declines by 6.2%. The smaller the particle size of the rubber powder, the shorter the pre-treatment time required to stabilize the physical properties of the rubberized asphalt. As the particle size of the rubber powder decreases, the crosslinking density after the reaction declines, and the viscosity of the rubberized asphalt at 180 °C also decreases. When the pre-treatment time is less than 8 h, the ductility at 5 °C increases; however, when the pre-treatment time exceeds 8 h, it first increases and then decreases. The softening point of the rubberized asphalt decreases, the penetration at 25 °C increases, and the elastic recovery at 25 °C decreases.

As previously discussed, the extent of de-crosslinking reactions in vulcanized rubber is closely related to the impregnation depth and uniformity of DD within the rubber phase. The deeper the impregnation and the more uniform the diffusion of DD, the more complete the de-crosslinking reaction. When the impregnation time and temperature are held constant, the impregnation and diffusion rates of DD are correlated with the specific surface area of the vulcanized rubber phase per unit mass. The larger the specific surface area, the deeper and more uniform the impregnation and diffusion of DD. For rubber powder-modified asphalt that has undergone pre-impregnation treatment, as the particle size of the rubber powder decreases, the amount of DD that permeates into the rubber powder is higher, and the diffusion is more uniform. At the same reaction time, the efficiency of DD in forming active free radicals and inducing de-crosslinking reactions is higher. This is reflected in the properties of rubberized asphalt, where the viscosity at 180 °C, softening point, and elastic recovery at 25 °C are lower, while the ductility at 5 °C and penetration at 25 °C are higher. Under conditions that meet the workability requirements for rubberized asphalt construction, the finer the rubber powder particle size, the shorter the reaction development time required.

The above results indicate that, during the preparation of high-rubber-content asphalt modified with rubber powder, DD undergoes a process of dissolution and dispersion, impregnation and diffusion, and the formation of active free radicals that dominate the de-crosslinking reactions. The pre-treatment time, temperature, and particle size of the rubber powder are closely related to the properties of rubberized asphalt. Longer DD pre-impregnation times, appropriate pre-impregnation temperatures, and smaller rubber powder particle sizes can significantly reduce the processing time for high-rubber-content asphalt modification, allowing the composite physical properties to meet the requirements for production and construction workability more rapidly. Excessively high pre-treatment temperatures can lead to the desulfurization and degradation of the rubber powder, thereby affecting the overall physical properties of the rubberized asphalt.

## 4. Conclusions

In this study, a solvent was employed as a dispersing medium to simulate the impregnation and diffusion process of the organic degradation agent diphenyl disulfide (DD) in vulcanized rubber, thereby comprehensively exploring the mechanism of action of DD in high-rubber-content asphalt modified with rubber powder. The key findings are summarized as follows:

(1) The mechanism of action of diphenyl disulfide (DD) in high-rubber-content asphalt modified with rubber powder was elucidated through the simulation of its impregnation and diffusion process in vulcanized rubber. This includes three stages: dissolution and dispersion, impregnation and diffusion, and the formation of active free radicals that dominate the de-crosslinking reactions.
(2) The dispersion of DD within rubber powder is most efficient under conditions of 12 h of impregnation, a temperature of 110 °C, and rubber powder particle sizes ranging from 160 to 180 µm. These conditions are crucial in achieving the rapid and uniform distribution of DD throughout the three-dimensional crosslinked network of the rubber powder, which significantly enhances the viscosity reduction of high-rubber-content asphalt.
(3) The adoption of pre-impregnated rubber powder in the asphalt modification process not only shortens the processing cycle but also enables the asphalt to reach superior physical properties in a more expedited manner compared to using untreated powder. This research provides actionable insights and a set of parameters that are valuable in guiding future industrial applications, thereby improving the overall efficiency and effectiveness of the asphalt modification process.

## Figures and Tables

**Table 1:** Raw material composition of vulcanized rubber SSR.

Raw Material	NR	SBR	ZnO	SA	CZ	NOBS	Sulfur
Composition (phr)	60	40	6	2.5	0.5	0.5	2.5

## Data Availability

The original contributions presented in this study are included in the article. Further inquiries can be directed to the corresponding authors.
